# Supplementary material for: Comparison of PB1-F2 Proximity Interactomes Reveals Functional Differences between a Human and an Avian Influenza Virus
Source: Viruses. 2023 Jan 24;15(2):328. doi: 10.3390/v15020328 (PMC9961899; doi:10.3390/v15020328)
Supplement: Supplementary file 1 [file viruses-15-00328-s001.zip › viruses-2106004-supplementary.pdf]

Table S1 - Mettier *et al*.

Protein ID	Gene Symbol	H7N1	H3N2	Gene Name	Protein class
P55011	SLC12A2	X	X	Solute carrier family 12 member 2	secondary carrier transporter
Q9NZI8	IGF2BP1	X	X	Insulin-like growth factor 2 mRNA-binding protein 1	RNA metabolism protein
P05165	PCCA	X	X	Propionyl-CoA carboxylase alpha chain, mitochondrial	ligase
Q15020	SART3	X	X	Squamous cell carcinoma antigen recognized by T-cells 3	RNA splicing factor
Q13442	PDAP1	X	X	28 kDa heat- and acid-stable phosphoprotein	-
P20020	ATP2B1	X	X	Plasma membrane calcium-transporting ATPase 1	primary active transporter
P02786	TFRC	X	X	Transferrin receptor protein 1	metalloprotease
P08195	SLC3A2	X	X	4F2 cell-surface antigen heavy chain	-
P00533	EGFR	X	X	Epidermal growth factor receptor	transmembrane signal receptor
Q8WYP5	AHCTF1	X	X	Protein ELYS	scaffold/adaptor protein
O00541	PES1	X		Pescadillo homolog	RNA metabolism protein
Q86UE4	MTDH	X		Protein LYRIC	-
Q01082	SPTBN1	X		Spectrin beta chain, non-erythrocytic 1	actin or actin-binding cytoskeletal protein
P61604	HSPE1	X		10 kDa heat shock protein, mitochondrial	chaperonin
Q9UIQ6	LNPEP	X		Leucyl-cystinyl aminopeptidase	metalloprotease
Q9BTC8	MTA3	X		Metastasis-associated protein MTA3	homeodomain transcription factor
Q6P1J9	CDC73	X		Parafibromin	-
O94776	MTA2	X		Metastasis-associated protein MTA2	homeodomain transcription factor
Q96I25	RBM17	X		Splicing factor 45	RNA splicing factor
Q99613	EIF3C	X		Eukaryotic translation initiation factor 3 subunit C	translation initiation factor
Q86VM9	ZC3H18	X		Zinc finger CCCH domain-containing protein 18	-
Q641Q2	WASHC2A	X		WASH complex subunit 2A	scaffold/adaptor protein
O95400	CD2BP2	X		CD2 antigen cytoplasmic tail-binding protein 2	RNA splicing factor
Q9UJU6	DBNL	X		Drebrin-like protein	scaffold/adaptor protein
Q92692	NECTIN2	X		Nectin-2	-
Q6NZI2	CAVIN1	X		Caveolae-associated protein 1	membrane traffic protein
Q96TA1	FAM129B	X		Protein Niban 2	-
Q99733	NAP1L4	X		Nucleosome assembly protein 1-like 4	chromatin/chromatin-binding, or -regulatory protein
P51116	FXR2	X		Fragile X mental retardation syndrome-related protein 2	translation factor
Q7Z478	DHX29	X		ATP-dependent RNA helicase DHX29	RNA helicase
P55265	ADAR	X		Double-stranded RNA-specific adenosine deaminase	RNA metabolism protein
P61011	SRP54	X		Signal recognition particle 54 kDa protein	RNA metabolism protein
Q05682	CALD1	X		Caldesmon	non-motor actin binding protein
Q92598	HSPH1	X		Heat shock protein 105 kDa	Hsp70 family chaperone
B5ME19	EIF3CL	X		Eukaryotic translation initiation factor 3 subunit C-like protein	translation initiation factor
Q13330	MTA1	X		Metastasis-associated protein MTA1	homeodomain transcription factor
Q9Y4E1	WASHC2C	X		WASH complex subunit 2C	scaffold/adaptor protein
Q9H6Z4	RANBP3	X		Ran-binding protein 3	scaffold/adaptor protein
Q96RT1	ERBIN	X		Erbin	scaffold/adaptor protein
P20810	CAST	X		Calpastatin	protease inhibitor
Q9H0U4	RAB1B	X		Ras-related protein Rab-1B	small GTPase
Q96AE4	FUBP1	X		Far upstream element-binding protein 1	RNA metabolism protein
Q13435	SF3B2	X		Splicing factor 3B subunit 2	RNA splicing factor
Q04637	EIF4G1	X		Eukaryotic translation initiation factor 4 gamma 1	translation initiation factor
P13861	PRKAR2A	X		cAMP-dependent protein kinase type II-alpha regulatory subunit	kinase modulator
Q9NYB9	ABI2	X		Abl interactor 2	scaffold/adaptor protein
Q9BY77	POLDIP3	X		Polymerase delta-interacting protein 3	RNA metabolism protein
Q14978	NOLC1	X		Nucleolar and coiled-body phosphoprotein 1	-
Q99426	TBCB	X		Tubulin-folding cofactor B	chaperone
Q8TEQ6	GEMIN5	X		Gem-associated protein 5	-
P00450	CP		X	Ceruloplasmin	oxidase
Q13813	SPTAN1		X	Spectrin alpha chain, non-erythrocytic 1	actin or actin-binding cytoskeletal protein
Q02952	AKAP12		X	A-kinase anchor protein 12	scaffold/adaptor protein
P35658	NUP214		X	Nuclear pore complex protein Nup214	transporter
Q9NUL3	STAU2		X	Double-stranded RNA-binding protein Staufen homolog 2	-
P29317	EPHA2		X	Ephrin type-A receptor 2	transmembrane signal receptor
Q04917	YWHAH		X	14-3-3 protein eta	scaffold/adaptor protein
P49792	RANBP2		X	E3 SUMO-protein ligase RanBP2	scaffold/adaptor protein
Q15637	SF1		X	Splicing factor 1	RNA splicing factor
Q12913	PTPRJ		X	Receptor-type tyrosine-protein phosphatase eta	protein phosphatase
E9PAV3	NACA		X	Nascent polypeptide-associated complex subunit alpha, muscle-specific form	basic helix-loop-helix transcription factor
P61981	YWHAG		X	14-3-3 protein gamma	scaffold/adaptor protein
O43396	TXNL1		X	Thioredoxin-like protein 1	oxidoreductase
Q9UPN7	PPP6R1		X	Serine/threonine-protein phosphatase 6 regulatory subunit 1	phosphatase modulator
Q96RQ3	MCCC1		X	Methylcrotonoyl-CoA carboxylase subunit alpha, mitochondrial	ligase
Q9NZM1	MYOF		X	Myoferlin	-
P81605	DCD		X	Dermcidin	-
Q13596	SNX1		X	Sorting nexin-1	scaffold/adaptor protein
P46060	RANGAP1		X	Ran GTPase-activating protein 1	GTPase-activating protein
Q14966	ZNF638		X	Zinc finger protein 638	RNA processing factor
P31947	SFN		X	14-3-3 protein sigma	scaffold/adaptor protein
P02545	LMNA		X	Prelamin-A/C	-
Q5VZ89	DENND4C		X	DENN domain-containing protein 4C	guanyl-nucleotide exchange factor
P62820	RAB1A		X	Ras-related protein Rab-1A	small GTPase
Q9Y5Y0	FLVCR1		X	Feline leukemia virus subgroup C receptor-related protein 1	secondary carrier transporter
P20930	FLG		X	Filaggrin	cytoskeletal protein
P05556	ITGB1		X	Integrin beta-1	integrin
P02788	LTF		X	Lactotransferrin	transfer/carrier protein
Q9P2E9	RRBP1		X	Ribosome-binding protein 1	scaffold/adaptor protein
P29966	MARCKS		X	Myristoylated alanine-rich C-kinase substrate	-
P55327	TPD52		X	Tumor protein D52	-
P01040	CSTA		X	Cystatin-A	protease inhibitor
P14923	JUP		X	Junction plakoglobin	-
P15924	DSP		X	Desmoplakin	intermediate filament binding protein
O00763	ACACB		X	Acetyl-CoA carboxylase 2	-

Figure S1 - Mettier *et al*.

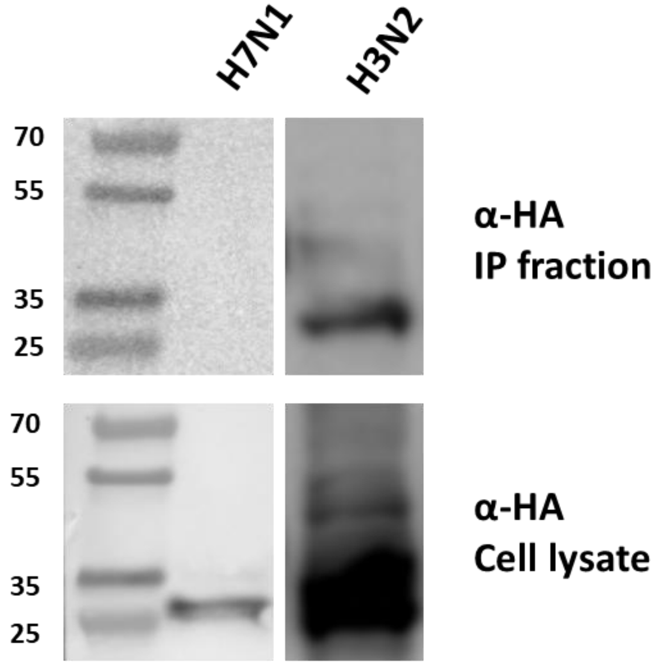

Figure S1 : Western blot analysis of the PB1-F2 – 14-3-3 protein interaction after immunoprecipitation assay. Cells were transiently transfected with constructs allowing the expression of HA-tagged YWHAH protein together with PB1-F2. Immunoprecipitations (IP) were performed with an anti-PB1-F2 antibody.
